# Breeding of medicinal and essential oil crops in VILAR: achievements and prospects

**DOI:** 10.18699/VJ21.048

**Published:** 2021-07

**Authors:** I.N. Korotkikh, D.N. Baleev, A.I. Morozov, P.G. Mizina, N.I. Sidelnikov

**Affiliations:** All-Russian Research Institute of Medicinal and Aromatic Plants, Moscow, Russia; All-Russian Research Institute of Medicinal and Aromatic Plants, Moscow, Russia; All-Russian Research Institute of Medicinal and Aromatic Plants, Moscow, Russia; All-Russian Research Institute of Medicinal and Aromatic Plants, Moscow, Russia; All-Russian Research Institute of Medicinal and Aromatic Plants, Moscow, Russia

**Keywords:** medicinal and essential oil plants, breeding, variety, breeding methods, лекарственные и эфирномасличные растения, селекция, сорт, методы селекции

## Abstract

This review discusses the main methods of breeding material development, the current state, problems and prospects for medicinal and essential oil plants breeding. The relevance of this area has especially increased due to the sanctions, the resulting shortage of medicinal plants and their low quality, which does not meet the requirements of the pharmaceutical industry. To produce a stable plant raw material base, it is necessary to actively develop a breeding process to create new highly productive varieties of medicinal plants resistant to biotic and abiotic environments. In breeding with the use of modern molecular biological methods, related species and generic complexes of the All-Russian Research Institute of Medicinal and Aromatic Plants (VILAR) collection can be involved, where there is extensive original genetic material of medicinal, essential oil, rare and endangered species. In the breeding of medicinal and essential oil crops, traditional methods of individual and individual-family selection, polyploidy, chemical mutagenesis and a combination of methods to obtain original breeding material are still promising. VILAR has created more than 90 varieties of medicinal and essential oil crops, most of which have been approved for use throughout the Russian Federation.

## Abstract (ru)

Рассмотрены методы создания селекционного материала, а также современное состояние, проблемы и перспективы селекции лекарственных и эфирномасличных растений в Российской Федерации. Актуальность данного направления особенно возросла в связи с объявленными санкциями, возникшим дефицитом лекарственного растительного сырья и низким его качеством, не удовлетворяющим требованиям фармацевтической индустрии. Для формирования устойчивой сырьевой базы необходимо создание новых высокопродуктивных, устойчивых к воздействию биотических и абиотических факторов среды сортов лекарственных растений с применением современных молекулярно-биологических методов в селекции. В этой связи большой интерес представляют коллекции Всероссийского научно-исследовательского института лекарственных и ароматических растений, в которых имеется уникальный генетический материал лекарственных и эфирномасличных, в том числе редких и исчезающих видов растений. В селекции лекарственных и эфирномасличных культур все еще перспективны традиционные методы индивидуального и индивидуально-семейного отбора, полиплоидии, химического мутагенеза и сочетание методов для получения оригинального селекционного материала. В институте создано более 90 сортов лекарственных и эфирномасличных культур, большинство которых допущены к использованию на всей территории РФ.

## Introduction

Currently, about 350,000 species of flowering plants have been described, but not every one of them is sufficiently studied to be called medicinal. According to the definition of the Great Medical Encyclopedia, medicinal plants are plants used as a source of medicinal plant raw material and medicinal products of natural origin^1^. Medicinal plant raw material is either fresh or dried plants, or their parts (grass, leaves, flowers, fruits, seeds, bark, buds, roots, rhizomes, bulbs, tubers, corms and others) used for the production of herbal medicines. Herbal medicines include fatty oils, essential oils, resins, balms, extracts, tinctures, aqueous extracts, and individual bioactive substances (BS) or their mixtures^2^. These drugs are recommended for the treatment and prevention of almost the entire spectrum of diseases.

The Great Medical Encyclopedia. Ed. by B.V. Petrovsky. 3rd edn. Vol. 12. Available at: URL:https://xn--90aw5c.xn--c1avg/


Russian State Pharmacopoeia. 14 edn. GMP.1.5.1.0001.15. Medicinal plant raw materials. Available at: URL: https://pharmacopoeia.ru/ofs-1-5-1-0001-15-lekarstvennoe-rastitelnoe-syre/


The chemical composition of medicinal plants and plantderived BS is extremely complex. In order to estimate which group of BS has a particular effect, All-Russian Research Institute of Medicinal and Aromatic Plants (VILAR) carries out a complex of chemical and pharmacological studies on extraction, fractionation, purification, isolation of BS from each studied object and determination of their specific activity. It is important that the species of medicinal plants growing in different botanical and geographical zones may contain the same groups of BS allowing in the absence of some necessary species to use alternative medicinal plants and obtain target substances. For example, the flavonoid rutin (vitamin P) which has been shown to strengthen the capillaries is found in varying quantities in the aerial part of the buckwheat (Fagopyrum esculentum Moench), the fruits of the chokeberry (Aronia melanocarpa (Michx.) Elliott) and black currant (Ribes nigrum L.), buds and fruits of Japanese sophora (Styphnolobium japonicum (L.) Schott), flowers and fruits of blood-red hawthorn (Crataegus sanguinea Pall.), different species of rose hips (Rosa L.), in the wood of the lower part trunk of Siberian larch and Gmelin larch (Larix sibirica Ledeb., Larix gmelinii (Rupr.) Kuzen). Another group of plant-derived BS are tannins, which are contained in the rhizomes of Bergenia crassifolia (L.) Fritsch, Bistorta off icinalis Delarbre, various types of potentilla (Potentilla L.), Sanguisorba off icinalis L., in the bark of various oak species (Quercus L.), viburnum (V iburnum opulus L.), bird cherry (Prunus padus L.), blueberry (Vaccinium myrtillus L.), multiple fruits of Alnus glutinosa (L.) Gaertn., leaves of Cotinus coggygria Scop. and Rhus coriaria L. The ability to accumulate cardiac glycosides of cardenolide and bufadienolide nature in tissues has been found in 20 species of medicinal plants belonging to 10 different families (Karpuk, 2011). 

The scientific program “From plant biochemistry to human biochemistry”, developed by VILAR, allows to study the biosynthesis of plant BS and purposefully use them for the improvement of people’s health, which is consistent with the goal of the state drug policy: to provide the population with affordable and high-quality medicines, including plant-derived ones, in a timely manner (Ulumbekova, Kalashnikova, 2018). Currently, their proportion occupies about 30 % of all drugs (Shirokova, 2013). And in this aspect, making a sustainable resource base of medicinal plants for pharmaceutical industry is of current interest. Among the set of tasks to solve this issue, the creation of new high-yielding varieties of medicinal and aromatic plants, resistant to the effects of biotic and abiotic factors, and the development of agricultural technologies for their cultivation is the most important.

## Modern approaches for breeding of medicinal plants

Biotechnology and molecular biology are used along with traditional breeding methods to create new varieties of medicinal plants. In such a case, the main goal is to increase both the yield of medicinal plant raw material and the content of certain secondary metabolites. Improved genotypes are important to increase the profitability of the production of high-quality medicinal plant materials.

Information on the genetic diversity and inheritance of the selected traits is one of the conditions for effective plant breeding (Wagner et al., 2005). The development of molecular biology methods opens many new possibilities for plant breeders to solve complex problems they face in traditional breeding process. Most modern research on medicinal plants focuses on the study of their genetic variability using DNA markers (Run et al., 2020). New high-yielding cultivar of Perilla frutescens L. was created using data of genome-wide sequencing and SNP analysis (Shen et al., 2017). At the same time, very little is known regarding the approaches to medicinal plants improving based on the molecular mechanisms of metabolite biosynthesis (Máthé, 2015). For example, several structural genes associated with the biosynthesis of flavonoids in gentian were isolated and characterized (Nakatsuka et al., 2008; Shimada et al., 2009). Wagner et al. (2005) showed the phenomenon of monogenic inheritance of the (–)-α-bisabolol and chamazulene content in chamomile. The greatest success has been achieved in Artemisia annua L. breeding, which produces the important sesquiterpene lactone called artemisinin (Graham et al., 2010; Townsend et al., 2013).

In vitro tissue cultures are often used in the breeding of medicinal plants (Máthé, 2015). Effective systems for cultivation and regeneration of tissues including the cultivation of callus, anthers, and protoplasts, have been created for some species such as Echinacea purpurea (L.) Moench, Dendrobium candidum Wall., Aristolochia contorta Bunge, Centella asiatica L. and Curcuma wenyujin Y.H. Chen. (Wang et al., 2020). Biotechnology techniques play an important role in the conservation of some medicinal plants – in particular endangered species. In spite of the number of problems, there are significant prospects for future development of this field of research.

## Varieties of medicinal plants created by VILAR and prospects for their industrial use

The institute has collected and created an original and unique genetic material base of medicinal and aromatic (including rare and endangered) plant species. However, until recently, this material has not been studied properly. Morphotypes, closely related species and generic complexes can be involved in breeding using modern methods. For these purposes, collections of the genera Digitalis L., Echinacea Moench, Origanum [Tourn.] L., Atropa L., Tanacetum L. and Mentha L. have been set up in VILAR in the last decade. The development of modern approaches using cytological and molecular biological methods is very promising for the study of medicinal plants. Studies using various modern methods of chromosomal analysis at the early stages of ontogenesis (seedlings, cotyledons, and the first true leaves) make it possible to establish the cytogenetic characteristics of promising plant lines, which can then be included in the breeding process. Many species of medicinal plants have small chromosomes (up to 3 microns). The methods of selection and application of chromosomal markers were tested on Potentilla alba L. (Muravenko et al., 2003). In a paper by Samatadze et al. (2018) it was shown that the chromosomes of P. alba 2n = 28 are very small (0.88 to 1.7 μm) and have a similar morphology (based of monochrome staining).

**Fig. 1.**
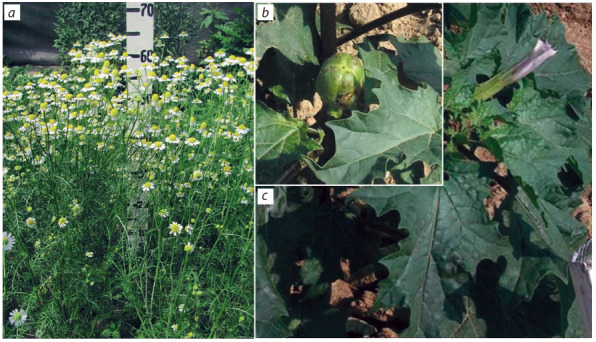
1. Matricaria chamomilla: variety Sibirskaya bisabololnaya (a); Datura stramonium: variety Besshipnyi, fruit (b), plant in the flowering phase (c).

In the breeding of medicinal and essential oil plants success is often achieved by a combination of methods for obtaining original breeding material (for example, exposure to mutagens in order to obtain polyploids for subsequent hybridization). Selection of polyploid forms obtained as a result of 0.2 % colchicine solution exposure has been successfully used on chamomile (Matricaria chamomilla L.). Among the three varieties recommended for cultivation in the Russian Federation, the variety Podmoskovnaya is an autotetraploid (2n = 36), and Nasten’ka and Sibirskaya bisabololnaya (Fig. 1, a) are diploids (2n = 18) (Khazieva et al., 2017). Polyploid variety Podmoskovnaya is distinguished from others by larger inflorescences, which are 1.5 times bigger than those in the standard (variety Azulena), an elongated peduncle and weak foliage – important factors for mechanized harvesting. At the same time, the effect of the same mutagen on seedlings of Datura stramonium L. did not cause a change in the ploidy level but led to a unique mutation: the absence of thorns in capsule fruits (see Fig. 1, b, c), which simplifies the harvesting of seeds and does not injure hands of plant collectors (Konon et al., 2012).

**Fig. 2.**
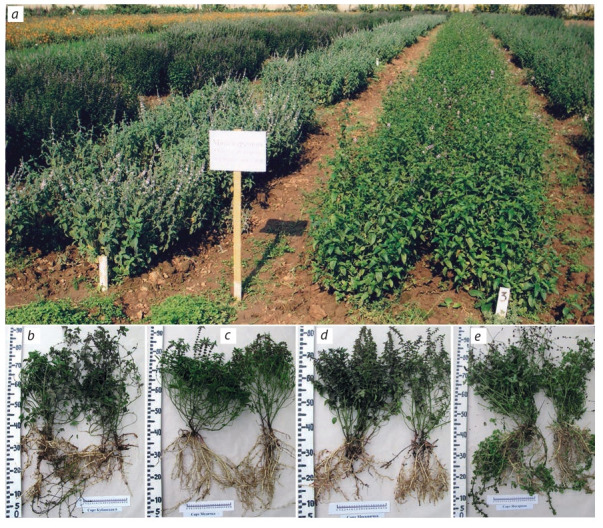
Mentha piperita: collection nursery (a); varietal plants: Kubanskaya 6 (b), Medichka (c), Moskvichka (d), Yantarnaya (e).

Polyploids were also used at the initial stage of peppermint breeding (Mentha×piperita L.) to obtain fertile plants and their generative offspring: when exposed to 0.025 % colchicine solution, a fertile allopolyploid was obtained (2n = 144). In mint breeding, a targeted selection of fertile forms with valuable traits (yield of leaves and above ground mass, content of essential oil and menthol) used for hybridization or to obtain generative offspring from free pollination was carried out. In this case, vegetative propagation and clonal selection were used at the stage of assessment and reproduction of elite plants selected in hybrid offspring. In interspecific hybridization, other species of mint (Mentha arvensis L. and M. sachalinensis Kudo) were used to increase the winter hardiness of hybrids: this is how the Prilukskaya 6, Yantarnaya, Kubanskaya 6, Lekarstvennaya 1, Lekarstvennaya 4, Moskvichka and Medichka varieties, which are widespread in industry, were created (Fig. 2). Due to winter hardiness and high productivity in various natural climatic conditions, these varieties were zoned for all regions of the Russian Federation and recommended for complex use, including for the production of essential oil and menthol (Morozov, 2018). The Aromatnaya mint variety was also isolated by clonal selection from a hybrid population; the essential oil of this variety has a mild taste and delicate aroma due to its low menthol content – an order of magnitude lower than that in the most other varieties (Morozov et al., 2012).

**Fig. 3.**
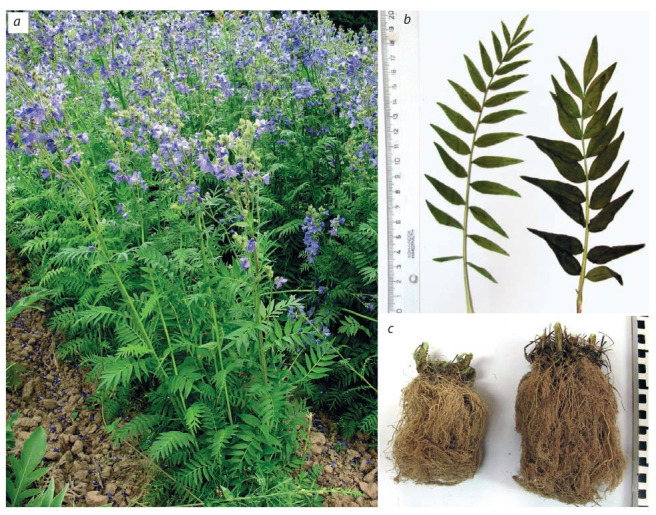
Polemonium coeruleum: variety Lazur’ (a); leaf and rhizome of diploid form (b, c, left) and tetraploid form (b, c, right).

In the breeding study carried out by Glazunova et al. (2020), chemical mutagenesis was also used to obtain polyploids (tetraploid, 2n = 4x = 36) of Polemonium coeruleum L. On the 2nd year of life polyploids were differed from diploid ones by sight (Fig. 3, b, c): tetraploids were undersized compact plants with a large number of peduncles. Moderate growth of the aboveground part contributes to the accumulation of active substances in the aboveground and underground parts; the volume and mass of the rhizome also increased.

**Fig. 4.**
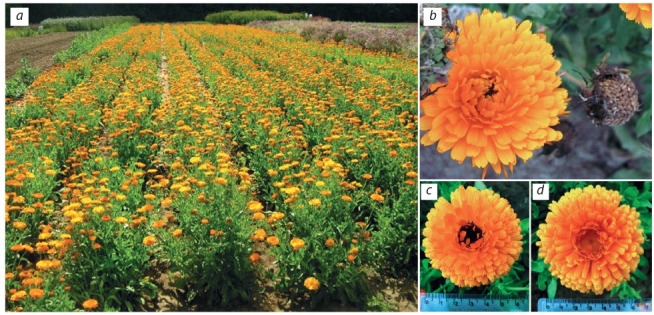
Calendula officinalis: nursery seed of the variety Zolotoe more (a); inflorescence and infruсtescenсe of the C. officinalis double inflorescences form (b); inflorescence of the varietal plant Rajskij sad (c) and Zolotoe more (d).

The breeding based on the species created by mutagenesis was carried out for Calendula off icinalis L. (Fig. 4, a). The most effective mutagens for C. off icinalis were 0.05 % diethyl sulfate and 0.08 % dimethyl sulfate. By selection for morphological characters, productivity and biologically active substances (in M1) and assessment for uniformity, distinctness and stability (in M2–3), new marigold varieties Zolotoe more (Fig. 4, d ) and Rajskij sad (Fig. 4, c) were developed. The yield of raw plant materials was 30–39 % higher compared to the standard variety Kal’ta, the content of extractives and total flavonoids content were increased by 13–21 and 29–43 %, respectively (Khazieva et al., 2016). The proportion of fractions suitable for mechanized sowing in the seed yield was increased: the fraction of hook-shaped seeds (up to 86 %), the fraction of ring-shared seeds (almost twofold) (see Fig. 4, b).

In the breeding of medicinal and aromatic plants, traditional methods of individual and individual-family selection are still promising, since most of these species were introduced into culture recently and are characterized by a high degree of polymorphism. Revealing the level of phenotypic variability and correlation of morphological and economically useful traits makes it possible to select the most productive morphotypes based on visual traits, easily taken into account. Breeding for productivity is carried out both for increasing the yield of raw plant materials and the content of BS

**Fig. 5.**
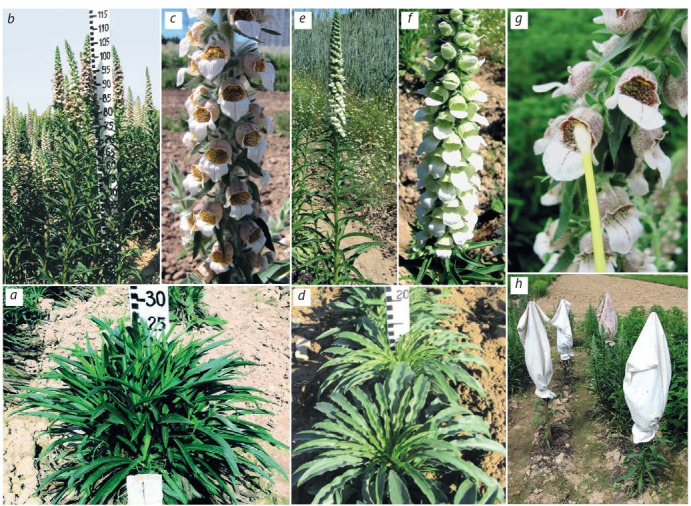
Digitalis lanata: plant variety Ritm, immature rosette of basal leaves of plants 1st year (a), generative shoots (b) and inflorescence (c) of plants 2nd year. D. lanata white-flowered form, immature rosette of basal leaves of plants 1st year (d), generative shoots (e) and inflorescence (f ) of plants 2nd year. Artificial pollination under the insulator (g); individually-insulated plant (h).

It should be noted that for the medicinal plants, the relationship between the yield of raw materials and the content of BS is a negative correlation value – this is because substances useful to humans are secondary metabolites used by plants for growth, development and adaptation to external factors. Therefore, sequential separate selection is mainly performed: initially, productive morphotypes are selected according to a complex of external characteristics (usually, the number and size of plant organs); after that, morphotypes with a high content of BS are selected. In accordance with the goals of breeding and the biological characteristics of particular plants, one or another method of selection and reproduction is used. For example, the breeding material of Digitalis lanata Ehrh. was created using forced self-pollination under the conditions of mechanical isolation (Fig. 5, g, h) and subsequent multiple systematic individual selection in 1st–3rd self-pollinated offspring (Korotkikh et al., 2013). Based on one of self‑pollinated offspring, the variety Ritm was created (see Fig. 5, a–c). In comparison with the standard variety Spectr, the yield of the raw material (leaves) of the new variety was 25–30 % higher and the adaptability to mechanized harvesting was improved due to the vertical deflection of the rosette leaves. The morphotypes were also differed in lanatoside content ranged from 0.22 to 0.65 %. As a result of repeated self-pollination, the original form of D. lanata was obtained. It was characterized by white flowers (see Fig. 5, d–f ) with a decorativeness as good as the widely known decorative varieties of D. purpurea L.; it even exceeds these varieties in flowering duration (41–47 days).

**Fig. 6.**
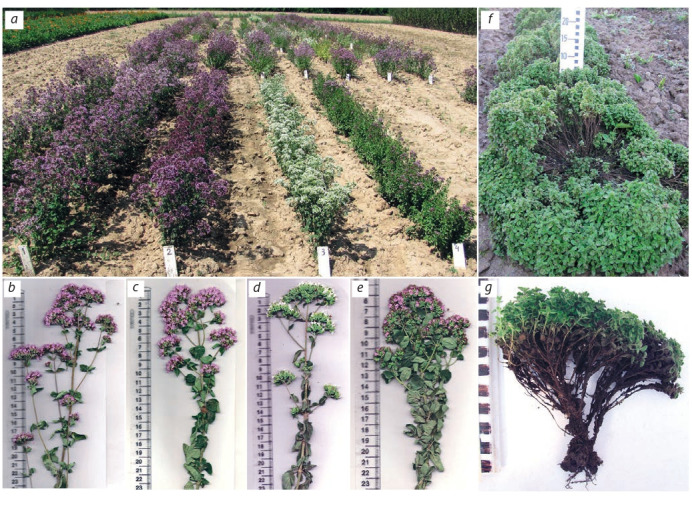
Origanum vulgare: collection nursery (a) and generative shoots of varieties Raduga (b), Slavnitsa (c), Zima (d), No. 12-06 (e); creeping form O. vulgare (f, g).

For breeding aromatic perennial herb oregano (Origanum vulgare L.), vegetative propagation by division of the rhizomes was used to select clones. In the development of young plants, two reproductive phases were noted (summer and fall); plants formed seeds in the current growing season and could be used in breeding and for preservation ex situ biological collection. Using the method of individual selection, we isolated the samples of oregano clones with high yields of raw materials and the content of essential oil (Korotkikh et al., 2015). The selection was carried out according to the height of the plants and the color of the flowers (Fig. 6, a). Tall forms including variety Raduga (see Fig. 6, b) were characterized by the maximum yield of raw plant materials. However, the maximum essential oil total harvest was possible from plants of medium height and low-growing plants due to the increased content of essential oil being between 0.8 and 2.4 times higher, which indicates their value for cultivation (see Fig. 6, c, e).

With repeated successive self-pollination of O. vulgare, the original creeping form was obtained (see Fig. 6, f, g), which does not form a typical rhizome with the aerial part approximately 10–12 cm tall and consists of three hundred or more thin succulent shoots. The content of essential oils corresponded to that in the initial form. Overall, this form could be recommended for food or decorative purposes.

The phytochemical study of essential oil samples of oregano (O. vulgare) varieties revealed that sesquiterpenes (β-elemene, α-copaen, β-caryophyllene, germacrene D, β-bisabolene, etc.) predominated in all varieties and their maximum content was found in the variety Zima (51 % in essential oil). The identification (or creation) of intraspecific chemotypes by the composition of the essential oil is relevant in connection with their specific pharmacological activity (antimicrobial, cytotoxic, analgesic, anti-inflammatory, antibacterial). The content of monoterpenes (α-thujene, α-pinene, sabinene, β-myrcene, α-terpinene, γ-terpinene, β-linalool, β-terpineol, borneol, etc.) in the variety Slavnitsa was 6 and 15 times higher than that in varieties Raduga and Zima, respectively. The highest content of phenolic compounds (thymol, methyl ether, thymol carvacrol) was found in the variety Raduga (Khazieva et al., 2019).

Supportive selection. Traditional breeding methods used in VILAR help in continuous breeding improvement with the involvement of already created varieties and primary seed production. However, multiple reproductions of the cultivated variety lead to accumulation of the low-value morphotypes resulting in decreased or lost stability of varietal indicators. Due to the instability of meteorological indicators, the frequency of drought, freezing, and soaking increases. Older varieties may not be adapted to such non-typical growing conditions.

**Fig. 7.**
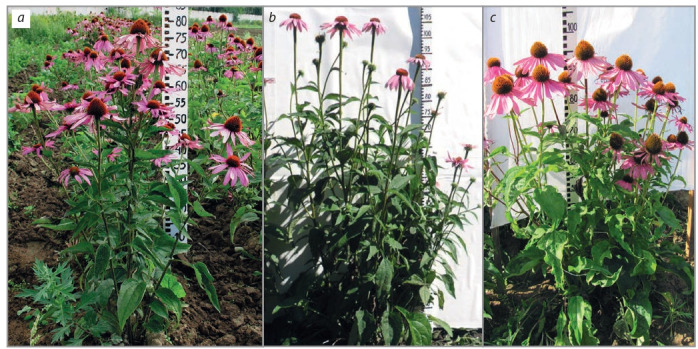
Echinacea purpurea: varietal plants Tanyusha (a), Yuzhanka (b), Severyanka (c).

Breeding of introduced species. With regard to introduced species, the aim of breeding is to increase not only valuable agronomic indicators (yield and quality of raw plant materials), but also indicators showing adaptation to regional conditions (seed productivity, duration of the growing season and winter hardiness). For example, only long-term acclimatization and mass selection in a cultivated population of Echinacea purpurea (L.) Moench made it possible to obtain high-quality seeds of local reproduction and subsequently to create national varieties, distribute and cultivate a new crop in Russia. Breeding of E. purpurea has been carried out in VILAR since 1996 and resulted in the first national variety Tanyusha (Fig. 7, a); in specific regional conditions of the North Caucasian branch of VILAR, the variety Yuzhanka was created (see Fig. 7, b). By individual selection using vegetative reproduction (initial form) and self-pollination (within the family), we obtained breeding material with stable productivity and adaptability which became the basis for a new variety Severyanka developed for the Non-Chernozem zone (see Fig. 7, c) (Korotkikh et al., 2019).

## Conclusion

Plant breeders of the VILAR and its branches have created more than 90 varieties of medicinal and aromatic plants using selection methods, intraspecific and interspecific hybridization, experimental polyploidy and mutagenesis for more than 70 years. A total 62 varieties, of which 17 varieties are protected by patents of the Russian Federation, were included in the “State Register of Breeding Achievements” in 2020 and approved for use on the territory of Russia. Collections of seeds and vegetative plants have been created and maintained to preserve varietal material.

Long-term studies have shown that in the breeding of medicinal and aromatic plants, the most promising is the complex study and use of the natural intraspecific variability of the crops. In recent years, there have been large-scale changes in molecular biology and information technology related to the study of genomes, transcriptomes, proteomes, small RNAs, epigenetics, gene editing and synthetic biology. Modern methods of breeding can involve morphotypes, closely related species and generic complexes. Collections of species promising for breeding and introduction are currently being formed at VILAR for these purposes. Therefore, the duration of the breeding cycle – which traditionally required 5–6 years for annual and biennial medicinal and aromatic crops, and 7–10 years for perennial crops – can be reduced if the studies are carried out year-round in laboratory and in greenhouses, making it less dependent on the duration of the growing season.

## Conflict of interest

The authors declare no conflict of interest.
